# Supplementary material for: Interplay between Energy Supply and Glutamate Toxicity in the Primary Cortical Culture
Source: Biomolecules. 2024 Apr 30;14(5):543. doi: 10.3390/biom14050543 (PMC11118065; doi:10.3390/biom14050543)
Supplement: Supplementary file 1 [file biomolecules-14-00543-s001.zip › biomolecules-2916495-supplementary.pdf]

biomolecules MDPI

Supplementary Materials for the Article "Interplay between Energy Supply and Glutamate Toxicity in the Primary Cortical Culture"

By Annette Vaglio-Garro [1,2]; Andrea Halasz [1]; Ema Nováková [1]; Andreas Sebastian Gasser [1]; Sergejs Zavadskis [1,2]; Adelheid Weidinger [1,2] and Andrey V. Kozlov [1,2,*]

Supplementary methods:

The size of the neurons and glia cells was determined in the pictures in where neurons were positive for MAP2, and glia cells were not. The images were analyzed in Image J (Version 2.14, National Institutes of Health, NIH, MD, USA), the images were exported as TIFF files, opened, and split from stack to images in Image J, the brightfield images were inverted, DAPI, MAP2 and brightfield were merge and copy to the system to be able to transfer the images into excel. The images were split again into three different channels (DAPI, MAP2 and brightfield). The size of the cellular body of neurons was obtained from the MAP2 images (**Figure S2.B,E,H**), filter/median 8 pixels was applied with a threshold of 70 to 255, particles were analyzed with the setting 50-infinity. The size of the cellular body of glia cells was obtained from brightfield images (**Figure S2.C,F,I**), filter/median 20 pixels was applied with a threshold of 20 to 255, particles were analyzed with the setting 50-infinity. The average and SEM were calculated, and the data was compared by Unpaired t-test by using GraphPad prism version 10 for Windows, GraphPad Software (Version 10.1.2, CA, USA). Figure S2.J,K,L show three examples of determination of the cellular body size of neurons lower than 100 μm^2, in compared to glial cells, which were higher than 1000 μm^2.

Supplementary Figures:

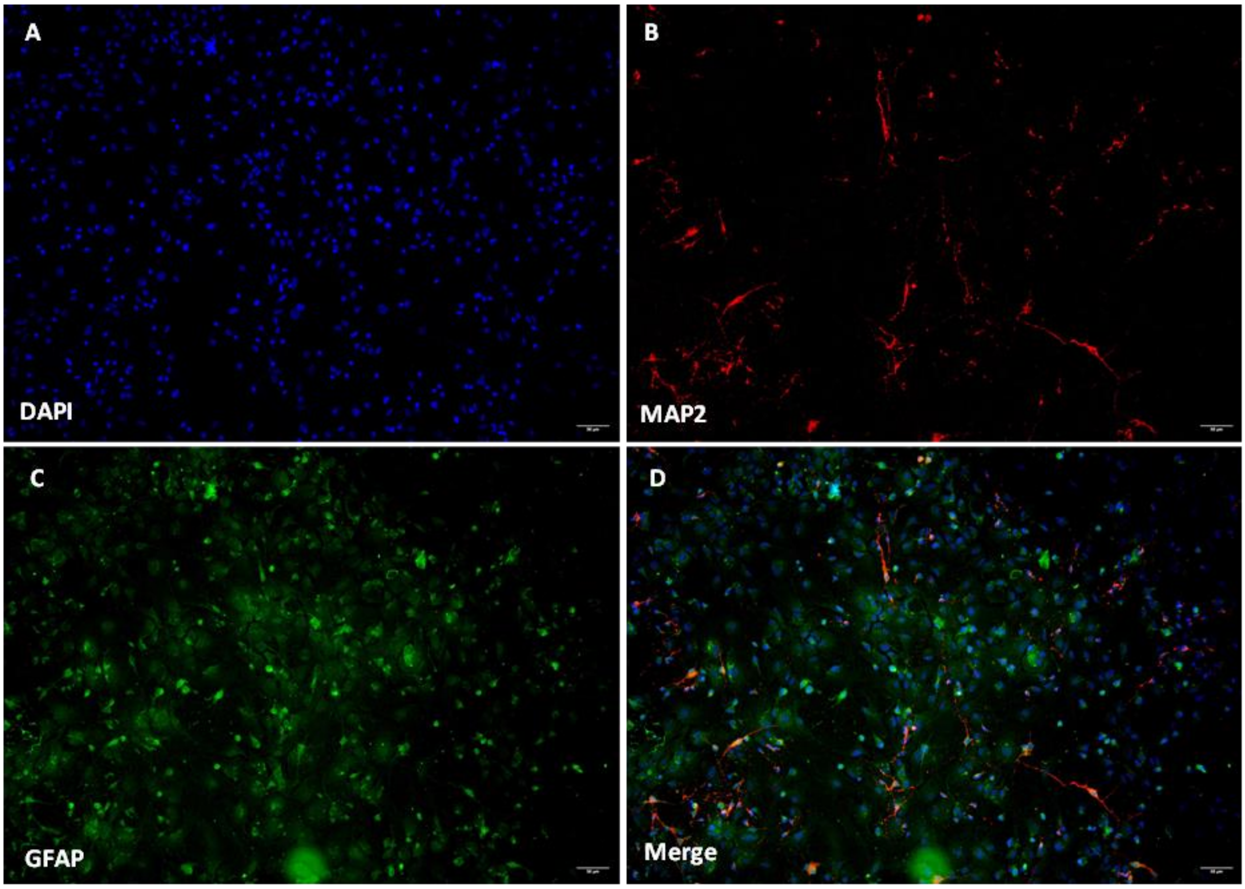

Supplementary figure s1. Primary cortical cells isolated from Sprague-Dawley rats. (A) Nuclei of all cells are identified with Dapi, (B) microtubules of neuronal cells with 594-conjugated MAP2 Rabbit PolyAb-Antibody, (C) glia cells are stained with 488-conjugated GFAP Mouse McAb-Antibody and (D) is the merge of all the signals. MAP2: microtu-bule-associated protein 2, GFAP: glial fibrillary acidic protein. Scale bar = 50 µm.

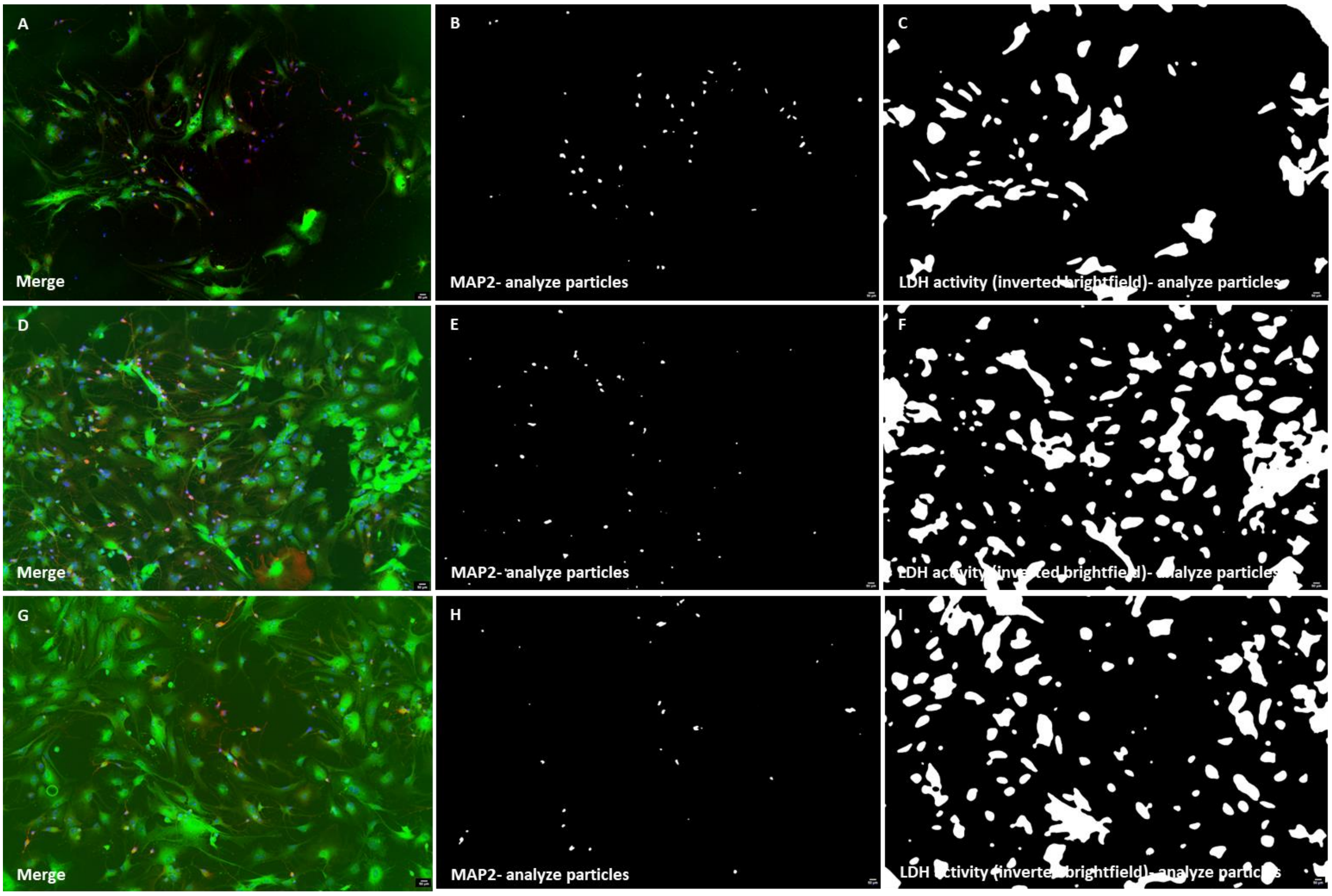

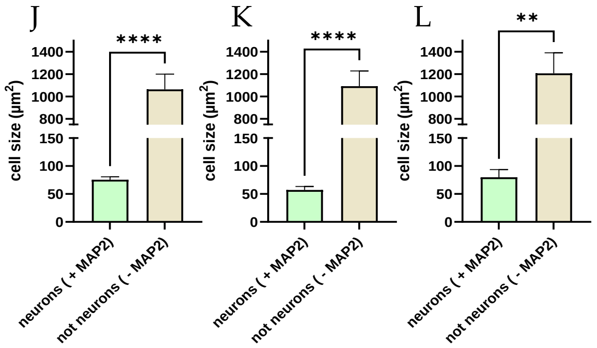

Supplementary figure s2. Primary cortical cells isolated from Sprague-Dawley rats cultured in glucose medium. (A,D,G) merge of all the signals images, (B,E,H) microtubules of neuronal cells with 594-conjugated MAP2 Rabbit PolyAb-Antibody, analyze particles images, (C,F,I) glia cells positive for LDH activity, analyze particles images of the inverted brightfield. (J,K,L) The quantification showed a lower size of cellular body area in neurons (<100 μm²) and larger in other cells (>1000 μm²). Data are presented as a mean ± SEM, n= 29-138. Statistical significance was determined using Unpaired t-test. **** p<0.0001, ** p<0.01. MAP2: microtubule-associated protein 2, LDH: lactate dehydrogenase. Scale bar = 50 μm.

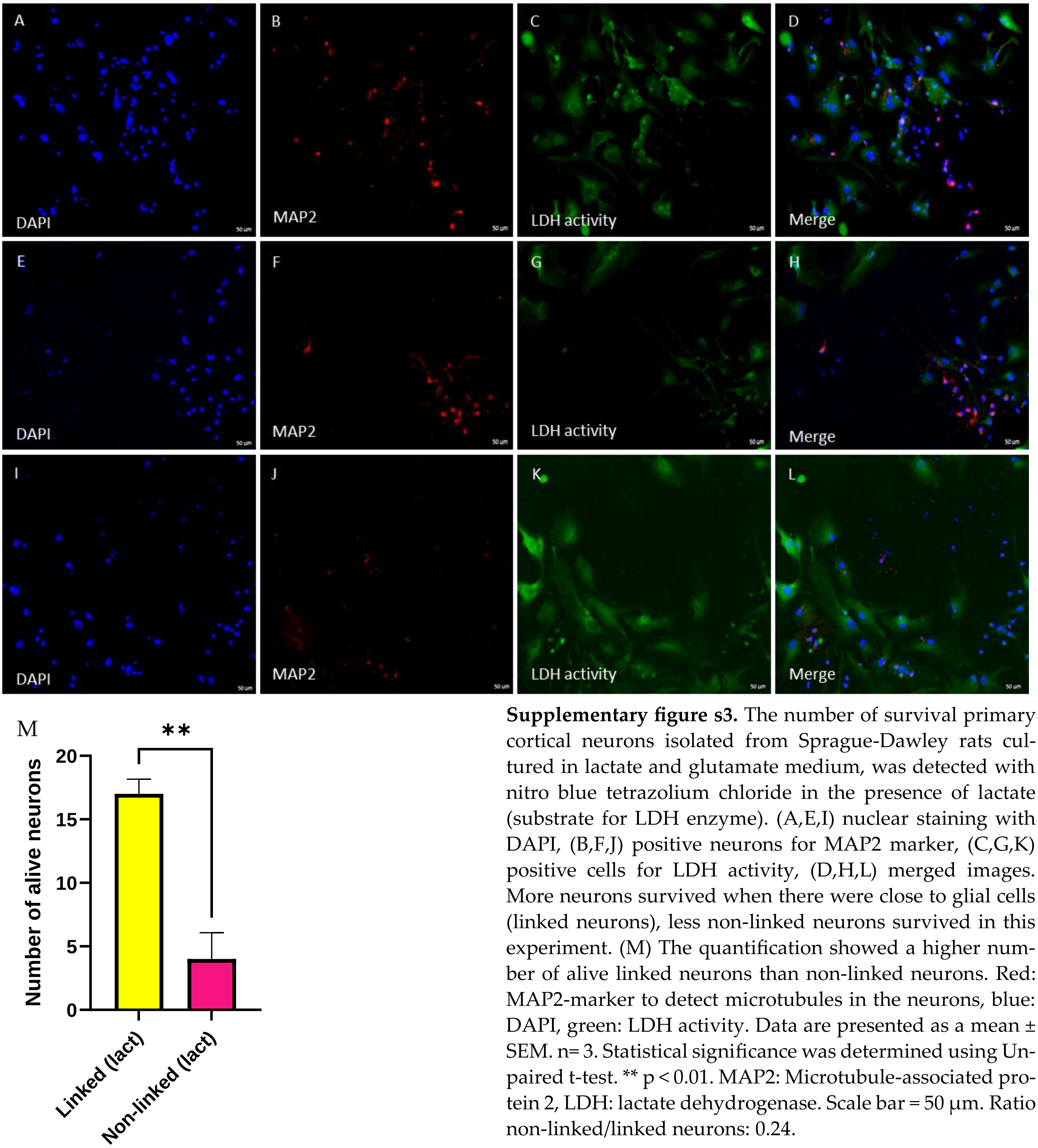

Supplementary figure s3. The number of survival primary cortical neurons isolated from Sprague-Dawley rats cultured in lactate and glutamate medium, was detected with nitro blue tetrazolium chloride in the presence of lactate (substrate for LDH enzyme). (A,E,I) nuclear staining with DAPI, (B,F,J) positive neurons for MAP2 marker, (C,G,K) positive cells for LDH activity, (D,H,L) merged images. More neurons survived when there were close to glial cells (linked neurons), less non-linked neurons survived in this experiment. (M) The quantification showed a higher number of alive linked neurons than non-linked neurons. Red: MAP2-marker to detect microtubules in the neurons, blue: DAPI, green: LDH activity. Data are presented as a mean ± SEM. n= 3. Statistical significance was determined using Unpaired t-test. ** $p < 0.01$. MAP2: Microtubule-associated protein 2, LDH: lactate dehydrogenase. Scale bar = 50 μm. Ratio non-linked/linked neurons: 0.24.

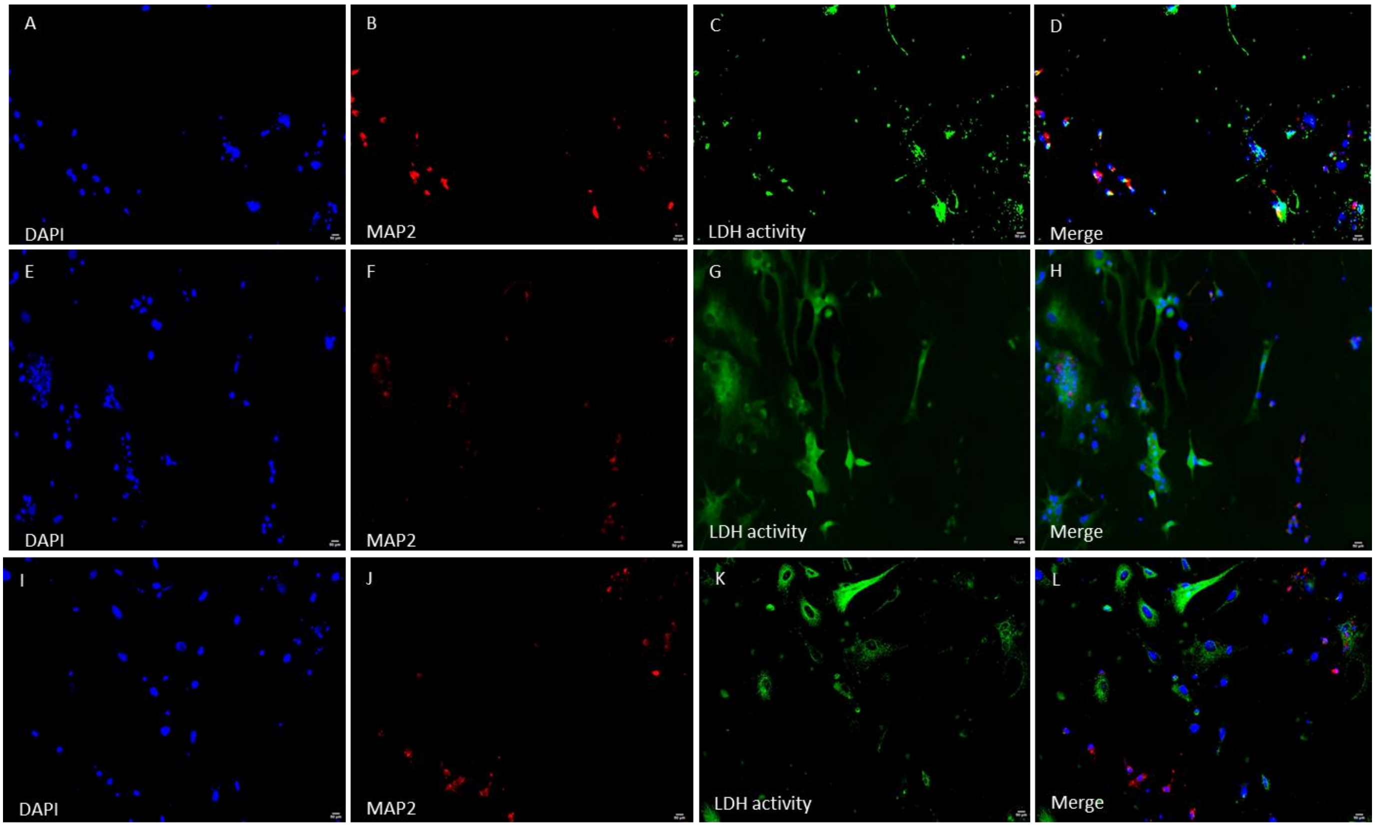

M

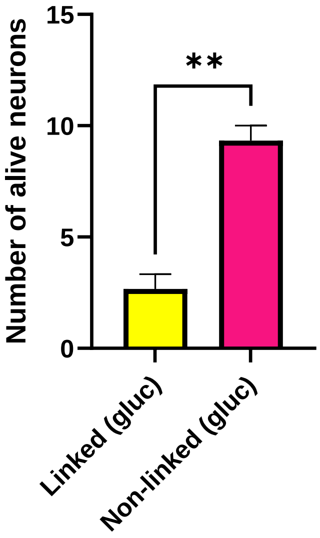

Supplementary figure s4. The number of survival primary cortical neurons isolated from Sprague-Dawley rats cultured in glucose and glutamate medium, was detected with nitro blue tetrazolium chloride in the presence of lactate (substrate for LDH enzyme). (A,E,I) nuclear staining with DAPI, (B,F,J) positive neurons for MAP2 marker, (C,G,K) positive cells for LDH activity, (D,H,L) merged images. Less neurons survived when there were close to glial cells (linked neurons), more non-linked neurons survived in this experiment. (M) The quantification showed a higher number of alive non-linked neurons than to linked neurons. Red: MAP2-marker to detect microtubules in the neurons, blue: DAPI, green: LDH activity. Data are presented as a mean ± SEM. n= 3. Statistical significance was determined using Unpaired t-test. ** $p < 0.01$. MAP2: Microtubule-associated protein 2, LDH: lactate dehydrogenase. Scale bar = 50 μm. Ratio non-linked/linked neurons: 3.48.
